# Case report of a functional evaluation of autonomy loss of a patient with TSP/HAM in PRM unit

**DOI:** 10.1186/1742-4690-12-S1-P94

**Published:** 2015-08-28

**Authors:** Barnay Jose Luis

**Affiliations:** 1 Centre Hospitalier Universitaire de Fort-de-France, Martinique (French West Indies

## 

An adult female patient with TSP/HAM for 30 years, started with neuropathic foot pain and impaired walking and a central neurological bladder dysfunction. She had abnormal permeability of the fallopian tubes treated by laparoscopic procedure, diabetes in the mother; there are three pregnancies and three parities. She was a housekeeper but she ceased due to the evolution of her illness. She was hospitalized in neurology because of repeated falls and pain in the lower left member with sciatica type of neuropathic pain. They did not found acute complications. She's been transferred in the PMR service for a functional assessment. We found uncomfortable Spasticity is predominant on rectus femoris, and sural triceps (Gastrocnemius> soleus). Walking is possible over a distance of 20 meters with a pair of crutches, the anteverted pelvis, a hip flexion deficit, a bilateral moderate mowing the passage of the step is possible by a toggle in the frontal plane and sway trunk in the sagittal plane forward. The others troubles was, low backpain and no urinary infection. We made walk test evaluations, before and after completion of anesthetic motor blocks on bilateral RECTUS FEMORIS and GASTROCNEMIUS. Walk tests of 10 meters before and after blocks remained stable. But the patient explains a significant decrease in pain and greater ease in walking. End of the day she complained of pain in her ankle. We proposed to her a botulinum toxin injection into selected targets, orthopedic shoes incorporating feet bullpen. A muscle building erector spinae muscles and abdominal cladding, bilateral paravertebral infiltration of corticosteroids for pain relief, and a stay of intensification in rehabilitation. Therapeutic motor block allows contemplated treatment of the spasticity in patients who also have a kind of muscle deficit at lower limbs, in the context of a comprehensive care.

